# Renshen-Baidu-San restores epithelial–immune crosstalk and drives type 2 immune repair in ulcerative colitis: an integrated multi-omics study

**DOI:** 10.3389/fimmu.2026.1777808

**Published:** 2026-03-11

**Authors:** Junyu Liu, Xinglong Liu, Wei Zhang, Li Song, Ying Xu, Yu Yang, Peiyu Xiong, Bo Jia

**Affiliations:** 1 School of Basic Medical Sciences, Chengdu University of Traditional Chinese Medicine, Chengdu, China; 2 Key Laboratory of Pathobiology, Ministry of Education, Nanomedicine and Translational Research Center, Electron Microscopy Center, China-Japan Union Hospital of Jilin University, Changchun, China; 3 Innovative Institute of Chinese Medicine and Pharmacy/Academy for Interdiscipline, Chengdu University of Traditional Chinese Medicine, Chengdu, China; 4 Hospital of Chengdu University of Traditional Chinese Medicine, Chengdu, China

**Keywords:** colon organoids, metabolomics, molecular docking, network pharmacology, Renshen-Baidu-San, ScRNA-seq, ulcerative colitis

## Abstract

**Background & Aims:**

Ulcerative colitis (UC) is a chronic inflammatory disease of the colonic mucosa characterized by recurrent flares and difficulty achieving sustained remission. Renshen-Baidu-San (BDS), a classical Chinese herbal prescription, has shown promising clinical benefit in relieving UC symptoms, but its underlying therapeutic mechanisms remain insufficiently defined. This study aimed to elucidate the multiple biological mechanisms underlying the therapeutic effects of BDS in UC.

**Methods:**

We utilized an integrated multi-omics and experimental approach, combining serum metabolomics, pharmacological network analysis, molecular docking, and single-cell RNA sequencing in a DSS-induced UC mouse model, supplemented by colon organoid experiments. Key findings were validated using histopathology, immunohistochemistry, immunofluorescence, quantitative RT-PCR, ELISA, and flow cytometry.

**Results:**

Serum metabolomics demonstrated that BDS modulates steroid- and lipid-derived metabolites, thereby influencing steroid hormone biosynthesis, bile acid turnover, and lipid pathways including arachidonic acid and linoleic acid metabolism. Network pharmacology based on serum-detected components further highlighted BDS’s regulatory effects on inflammatory signaling and cellular proliferation, while molecular docking identified stable and favorable protein-ligand interactions. Single-cell transcriptomics revealed that BDS corrected UC-induced epithelial–immune dysregulation, with Tuft and T-1 cell subclusters emerging as key responders through coordinated suppression of NF-κB–driven TNF-α signaling; ligand–receptor analysis indicated restoration of epithelial–immune communication. Colon organoid experiments corroborated mucosal repair, crypt structural recovery, Tuft cell expansion, and a shift toward a tissue-restorative type 2 immune profile, characterized by elevated IL-4 and IL-25 and reduced IL-13.

**Conclusion:**

Our findings indicate that BDS treatment in DSS-induced colitis is accompanied by alterations in metabolic pathways, epithelial-immune communication, and cell-type-specific transcriptional programs, which may be relevant to type 2 immune–mediated mucosal repair processes.

## 1 Introduction

Ulcerative colitis (UC) is a complex chronic inflammatory bowel disease characterized by persistent mucosal inflammation, clinically manifesting as abdominal pain, diarrhea, rectal bleeding, and mucopurulent discharge (1). This relapsing-remitting disorder involves multifaceted pathogenesis encompassing immune dysregulation, epithelial barrier dysfunction, and microbial dysbiosis. Epidemiological studies reveal a rapidly increasing global incidence of UC (2). Current standard treatments—such as aminosalicylates and biologic agents—focus on maintaining long-term remission but remain limited by low response rates and high relapse rates (3), underscoring the urgent need for more effective therapeutic options.

Tuft cells are a highly differentiated epithelial lineage broadly distributed throughout the gastrointestinal tract, respiratory tract, and pancreas (4). Although first described more than 60 years ago, their function remained elusive until recent studies redefined them as central regulators of intestinal immunity and repair, with impaired regenerative capacity and immune imbalance linked to UC pathogenesis (5–7). Specifically, gut-resident tuft cells sense microbial cues and secrete key cytokines—most notably interleukin-25—to maintain immune homeostasis, modulate inflammation, and preserve mucosal barrier integrity (8). Notably, under type 2 immune cytokines IL-4/IL-13 stimulation, tuft cells can proliferate and even dedifferentiate into stem-like cells during inflammatory injury, serving as an endogenous “reserve stem cell pool” to promote epithelial repair (9, 10). Moreover, by expressing doublecortin-like kinase 1 (DCLK1), tuft cells regulate inflammation-associated repair mediators, further enhancing epithelial regeneration (11, 12). Collectively, these multifaceted roles position tuft cells as pivotal players in both the inflammatory progression and tissue restitution of UC, underscoring their emerging importance in disease mechanism studies (8).

Renshen-Baidu-San (BDS) is a classical traditional Chinese medicine formulation composed of 12 herbal components (**Table 1**), with its earliest documented use tracing back to Qian Yi’s *Direct Formula for Children’s Drug Syndromes* during the Northern Song Dynasty. In traditional medicine, BDS is recognized for its therapeutic effects: replenishing qi, eliminating dampness, and modulating immune function (13, 14). Extensive clinical practice has demonstrated its efficacy in alleviating UC-associated symptoms including abdominal pain, diarrhea, and hematochezia (15), making it a widely adopted UC treatment in China. Compared with current UC therapies such as aminosalicylates, corticosteroids, immunomodulators, and biologics—which mainly suppress inflammation or immune overactivation (16)—BDS has been traditionally applied to improve mucosal healing and epithelial restoration (17). This highlights its potential complementary value in UC management. Our previous studies demonstrated that BDS effectively promotes intestinal epithelial cell proliferation and repairs mucosal barrier damage in DSS-induced colitis models (17, 18). Mechanistically, BDS could attenuation of oxidative stress through SOD activation and reduction of ROS/MDA levels, and rebalancing of pro-inflammatory cytokines via downregulation of TNF-α, IL-1β, and IL-6 (19). Despite these advances revealing partial regulatory pathways, the comprehensive mechanistic network underlying BDS’s therapeutic effects on epithelial repair in UC remains incompletely elucidated. In particular, the precise molecular targets and integrated signaling pathways governing its barrier-restorative properties require systematic investigation.

**Table 1:** The herbal composition of BDS formula.

Herbal medicine	Chinese name	Family	Part of used	Weight/g
*Panax ginseng* C. A. Mey.	Ren Shen	Araliaceae	Root and rhizome	9
*Glycyrrhiza uralensis* Fisch.	Gan Cao	Fabaceae	Root and rhizome	9
*Bupleurum chinense* DC.	Chai Hu	Apiaceae	Root	9
*Ligusticum sinense* Oliv.	Chuan Xiong	Apiaceae	Rhizome	9
*Peucedanum praeruptorum* Dunn	Qian Hu	Apiaceae	Root	9
*Platycodon grandiflorus* (Jacq.) A.DC.	Jie Geng	Campanulaceae	Root	9
*Notopterygium incisum* Ting ex H. T. Chang	Qiang Huo	Apiaceae	Root and rhizome	9
*Heracleum hemsleyanum* Diels	Du Huo	Apiaceae	Root	9
*Poria cocos* (Schw.) Wolf	Fu Ling	Polyporaceae	Outer dermis	9
*Citrus aurantium* L.	Zhi Ke	Rutaceae	Immature fruit	9
*Mentha canadensis* L.	Bo He	Lamiales	Aerial parts	2
*Zingiber officinale* Roscoe	Sheng Jiang	Zingiberaceae	Rhizome	3

The herbal name has been checked with MPNS (http://mpns.kew.org).

In this study, we integrated metabolomics, network pharmacology, molecular docking and single-cell transcriptomics to comprehensively characterize the bioactive constituents and metabolic signatures of BDS. Focusing on tuft cells and their downstream immune‐regulatory networks, we then validated these mechanistic insights in a colonic organoid model. Our findings offer mechanistic insights that support the clinical application of BDS in ulcerative colitis.

## 2 Materials and methods

### 2.1 Chemicals and reagents

HPLC grade acetonitrile, formic acid and methanol were purchased from Thermo Fisher Scientific Co., Ltd (Waltham, MA, USA); Ultrapure water was purified using UNIQUE laboratory multifunctional ultra-pure water system (Fujian, China); DSS (mw: 36–50 kDa) was purchased from MeilunBio Biological Technology Co., Ltd (Dalian, China); Collagenase I, II, and neutral protease were obtained from Sangon Biotech (Shanghai, China); The cell viability dyes Calcein AM (live), DRAQ7™ (dead) and RevertAid First Strand cDNA Synthesis Kit were purchased from Thermo Fisher Scientific Co., Ltd (Waltham, MA, USA); Qubit™ dsDNA HS Assay Kit was obtained from Beckman Coulter Co., Ltd (Brea, CA, USA); Enhanced Cartridge Reagent and BD Rhapsody™ WTA Amplification Kit were obtained from BD Biosciences Co., Ltd (San Jose, CA, USA); KAPA Library Quant Kit was obtained from Illumin, Inc. (San Diego, CA, USA); Matrigel^®^ Matrix for Organoid Culture was obtained from Corning Inc. (NY, USA); Intestinal tissue digestion solution and organoid growth medium was obtained from Aimingmed Technologies Co., Ltd. (Hangzhou, China); Trizol-bead-based RNA extraction kit was obtained from Genstone Biotech Co., Ltd. (Beijing, China); RevertAid First Strand cDNA Synthesis Kit was obtained from Thermo Fisher Scientific Co., Ltd (Waltham, MA, USA); TB Green^®^ Premix Ex Taq was obtained from TakaraBio Inc. (Beijing, China); Anti-DCAMKL1, anti-Ki67 antibodies and Goat Anti-Rabbit IgG H&L (Alexa Fluor^®^) were provided by Abcam (Cambridge, MA, USA); Anti-GATA3 antibody was provided by Proteintech Group (Wuhan, China); APC Anti-Mouse CD170 and FITC Anti-Mouse CD326 antibodies were obtained from Elabscience (Wuhan, China); Elisa kit of IL-4, IL-13 and IL-25 were purchased from Jianglai Biotechnology Co., Ltd (Shanghai, China).

### 2.2 Animals

Male SD rats (SPF grade, 180–220 g) and male C57BL/6 mice (SPF grade, 20–22 g) were provided by Chengdu Dashuo Experimental Animal Co., Ltd (Animal License No. SCXK (Chuan) 2020-030). All animals were housed under controlled conditions with free access to food and water, and acclimatized for one week prior to experiments. All experimental procedures were approved by the Animal Ethics Committee of Chengdu University of Traditional Chinese Medicine (Approval No. 2023040) and conducted in strict accordance with institutional guidelines. SD rats were orally administered BDS at a dose of 8.55 g/kg daily for seven consecutive days. On the seventh day, blood samples were collected 2 hours after the final administration.

C57BL/6 mice were randomly allocated into four groups (n=6 per group): Normal group (normal saline), Model group (3% DSS, w/v), Treatment group (BDS, 12.35 g/kg), and 5-ASA group (5-aminosalicylic acid, 200 mg/kg). To induce ulcerative colitis, all groups except the normal received 3% DSS (w/v) in drinking water for 7 consecutive days. During days 8-14, the Treatment group received daily oral gavage of 8.55 g/kg BDS, while the 5-ASA Treatment group was administered 200 mg/kg 5-ASA; Normal and Model groups received equivalent normal saline volumes. On day 15, all mice were euthanized under anesthesia pentobarbital sodium (50 mg/kg, i.p.), and colon tissues were harvested for subsequent experiments.

### 2.3 BDS and serum sample preparation

All herbal materials were provided by Chengdu Kangmei Pharmacy (Sichuan, China) and authenticated by Sichuan Limin Traditional Chinese Medicine Decoction Pieces Co., Ltd., meeting the quality standards specified in the 2020 Chinese Pharmacopoeia. The aqueous extract of BDS was prepared according to established methods (20). The herbal mixture was decocted for 1 hour, followed by filtration and concentration to 8.55 g/ml and 12.35 g/kg using a rotary evaporator. All prepared decoctions were stored at 4°C for subsequent use. The complete herbal composition is shown in **Table 1**. After 7 consecutive days of oral administration with 8.55 g/kg of BDS, blood samples were collected from SD rats 2 hours following the final dose. The blood was allowed to clot for 30 minutes at room temperature, followed by centrifugation at 3500 rpm for 15 min to obtain serum. The serum was subsequently heat-inactivated at 56°C for 30 min, filtered through a 0.22 μm membrane, and stored at -80°C until further use.

Prior to UPLC-Q-Exactive-MS/MS analysis, both BDS aqueous extract and serum samples were subjected to methanol pretreatment. Briefly, proteins were precipitated by adding 800 μl of methanol to 200 μl of serum (1:4, v/v). After vortexing for 30 seconds, the mixture was centrifuged at 15000 rpm, 4°C for 30 min and subsequently dried under nitrogen gas at 25°C. The residue was reconstituted in 200 μl of 70% methanol, followed by centrifugation at 15,000 rpm for 5 min. After microfiltration through a 0.22 μm membrane, 10 μl of the processed sample was injected for analysis. For quality control, 10 μl aliquots from each sample were pooled to create a composite QC sample.

### 2.4 UPLC-Q-exactive-MS/MS analysis conditions

The analytical methods and parameters were adapted from previously established protocols (21). Both the BDS aqueous extract and serum samples were analyzed using UHPLC Exactive system (Thermo Fisher Scientific) equipped with Q Exactive quadrupole-electrostatic field orbitrap high-resolution mass spectrometer. The chromatographic conditions were as follows: An ACQUITY UPLC BEH C_18_ column (100 mm × 2.1 mm, 1.7 μm; Waters, Milford, USA) was maintained at 40°C. The mobile phase consisted of (A) 2% acetonitrile in water with 0.1% formic acid and (B) acetonitrile with 0.1% formic acid, delivered at 0.40 mL/min with a 3 μl injection volume. The gradient program was: 0-0.1 min, 0-5% B; 0.1–2 min, 5-25% B; 2–9 min, 25-100% B; 9–13 min, 100% B; 13-13.1 min, 100-0% B; 13.1–16 min, 0% B. Mass spectrometric detection was conducted using electrospray ionization in both positive and negative modes (m/z 70-1050). Key parameters included: spray voltage 3500 V (positive) and 3000 V (negative), sheath gas 50 psi, auxiliary gas 13 psi, ion transfer tube temperature 450°C, stepped normalized collision energy 20-40–60 V, with MS resolution of 70,000 and MS/MS resolution of 17,500.

### 2.5 Identification of drug prototype components in serum

LC/MS raw data were pretreated and analyzed using Progenesis QI v3.0 (Waters Corporation, Milford, USA), generating a three-dimensional matrix (sample, metabolite, intensity). Internal standards and false positives were removed, followed by dereplication and peak pooling. Metabolites were identified using HMDB, TCMSP, and Majorbio Database. Retaining features detected in ≥80% of samples. Missing values were replaced with minima, and sum normalization was applied. QC samples with RSD>30% were excluded, and log10 transformation yielded the final matrix for subsequent analysis. For drug prototype component identification, the complete ion fragment data from the acquired mass spectra were matched to the Pubchem database and analyzed using MassFragment software to confirm drug prototype components in serum (DPCs).

### 2.6 Non-targeted metabolomic analysis

The R package “ropls” (Version 1.6.2) was used to perform principal component analysis (PCA) and orthogonal least partial squares discriminant analysis (OPLS-DA), and 7-cycle interactive validation evaluating the stability of the model. The metabolites with VIP>1, P<0.05 and FC>–1 were determined as significantly different metabolites based on the Variable importance in the projection (VIP) obtained by the OPLS-DA model and the p-value generated by student’s t test. P-values from univariate tests were adjusted for multiple comparisons using the Benjamini–Hochberg method (FDR), and adjusted P-values were used where indicated. Differential metabolites among two groups were mapped into their biochemical pathways through metabolic enrichment and pathway analysis based on KEGG database (http://www.genome.jp/kegg/). These metabolites could be classified according to the pathways they involved or the functions they performed. Enrichment analysis was used to analyze a group of metabolites in a function node whether appears or not. Python packages “scipy.stats” (https://docs.scipy.org/doc/scipy/) was used to perform enrichment analysis to obtain the most relevant biological pathways for experimental treatments.

### 2.7 Network pharmacology study

The potential targets of DPCs were retrieved from the TCMSP Database (https://tcmspw.com/) and SwissTargetPrediction databases (http://www.swisstargetprediction.ch/). The obtained targets were then standardized to human gene symbols using the UniProt database (https://www.UniProt.org/). Disease-related targets of ulcerative colitis (UC) were collected from the GeneCards (https://www.genecards.org), OMIM (https://omim.org/) and DisGeNET (https://disgenet.com/) databases. The overlapping targets between DPCs and UC were identified as potential therapeutic targets for further analysis. The overlapping targets between DPCs and UC were imported into the STRING database (https://www.string-db.org/) to construct a protein-protein interaction (PPI) network. The network was analyzed using Cytoscape 3.10.3 software, and the intersection targets subsequently screened by the CytoNCA, Mcode, and Cytohubba plugins were identified as core targets for molecular docking. Subsequently, a comprehensive “compound-disease-target” network was visualized to elucidate their interactions.

### 2.8 Molecular docking

To further identify DPCs with high binding affinity among the core targets, molecular docking was performed using AutoDock Vina (v1.1.2). The ligands were identified through metabolomic analysis, while the receptors were the core targets validated by our network pharmacology approach. The three-dimensional structures of these ligands were retrieved from the PubChem database, and the crystal structures of the target proteins were obtained from the RCSB Protein Data Bank. Prior to docking, the protein structures were preprocessed with PyMOL (v3.1.3). A binding energy value of ≤ −5 kcal/mol was set as the criterion for defining significant binding interactions, which were visualized using Ligplot (v2.2.9) to generate two-dimensional diagrams.

### 2.9 Single-cell suspension preparation from mouse colon tissue

Colon tissues were longitudinally opened, washed with DPBS, and cut into 3–5 mm segments in a sterile dish before further mincing in a 15 ml tube. After two additional DPBS washes until the supernatant was clear, the tissue fragments were digested in pre-warmed dissociation enzyme I (collagenase I + collagenase II + neutral protease) at 37°C with shaking for 8 min. The digestate was filtered through a 70 μm strainer, and the supernatant was centrifuged (500 rcf, 5 min, 4°C) to pellet cells. After red blood cell lysis using 1 ml lysis buffer for 5 min at room temperature, and DPBS quenching, cells were resuspended in Stain Buffer for counting. Residual tissue was subjected to a second digestion using dissociation enzyme II (0.25% Trypsin-EDTA + neutral protease). Finally, cells were resuspended in Stain Buffer, stained with Calcein AM and DRAQ7™ to assess viability/counts via BD Rhapsody™ Scanner (BD Biosciences, USA), and loaded onto an 8-channel microchip with pre-cooled Sample Buffer for single-cell capture using BD Rhapsody™ HT Xpress (BD Biosciences, USA).

### 2.10 Single-cell library preparation and sequencing

All procedures were strictly performed according to the BD Rhapsody™ Whole Transcriptome Analysis (WTA) and Library Construction Standard Protocol. Briefly, after cell capture using magnetic beads, beads were lysed and washed with pre-chilled Bead Wash Buffer. cDNA synthesis was performed using the BD Rhapsody™ WTA Amplification Kit (San Jose, CA, USA), including reverse transcription, exonuclease I treatment, random primer extension (RPE), and PCR amplification. The RPE products were purified using AMPure XP beads, followed by WTA Index PCR to add sequencing adapters. Sample Tag PCR was performed to barcode libraries. Library quality was assessed using the Qubit™ dsDNA HS Assay Kit (Brea, CA, USA), followed by quantification with the KAPA Library Quant Kit (San Diego, CA, USA). Qualified libraries were sequenced on the NovaSeq XPlus platform (Illumina, USA).

### 2.11 Data preprocessing and dimensionality reduction

Sequencing data were preprocessed using the BD Rhapsody Pipeline (v2.0) with default parameters to generate a single-cell expression matrix. The pre-built mouse GENCODE M31 whole transcriptome (WTA) reference obtained from the BD Biosciences website was used for alignment. Downstream analyses were performed using Scanpy (v1.9.6). For quality control, we retained only cells expressing at least 200 genes and genes detected in a minimum of 3 cells. Cells exhibiting an unusually high number of expressed genes (>4500) or a high percentage of mitochondrial gene counts (>30%) were excluded. Gene expression values were normalized using default parameters to obtain a normalized expression matrix. Highly variable genes were selected based on Scanpy’s default criteria (min_mean, 0.0125; max_mean, 3; min_disp, 0.5). Principal component analysis was applied, and the top 30 principal components were used for Uniform Manifold Approximation and Projection (UMAP) visualization and Leiden clustering.

### 2.12 Cell type annotation

After the clustering, SingleR (v2.2.0) was utilized for cell type annotation. All cells were preliminarily categorized into five major types: endothelial cells, epithelial cells, macrophages, B cells, and TNK cells (the dataset used for annotation comes from the Single Cell Portal database from Broad Institute, with the dataset numbered SCP2038) (22);. Subsequently, epithelial cells and TNK subgroups were extracted separately for further re-clustering analysis. These subpopulations subsets were re-clustered and annotated based on well-established marker genes from public databases and published studies (23). Due to pooling prior to library construction, cluster-level annotation confidence was prioritized to avoid potential doublet-driven misclassification.

### 2.13 Identification of differentially expressed genes and enrichment analysis

Differential gene expression analysis was performed using Scanpy (v1.9.6) to compare cell populations across experimental groups. Genes with |log_2_FC| > 1 and adjusted p-value < 0.05 were considered differentially expressed genes (DEGs). Enrichment analysis was conducted on these DEGs, retaining terms with false discovery rate (FDR) < 0.05 for visualization. Gene Set Enrichment Analysis (GSEA), implemented via GSEApy (v1.1.3) was applied to identify coordinated expression changes in predefined gene sets. All analyses used default parameters unless specified.

### 2.14 Cell communication analysis

CellChat (v2.1.0) with default parameters was used to analyze cell-cell communication. Based on cell types and sample-specific ligand-receptor interactions from its built-in mouse database, we visualized communication networks and generated heatmaps (24).

### 2.15 Colonic crypt isolation and organoid culture

The mouse colon was longitudinally opened, segmented into 4–5 mm pieces, and washed 15–20 times until the effluent was clear. Tissue fragments were digested on ice for 30 min in intestinal tissue digestion solution (Hangzhou, China) with intermittent agitation, followed by centrifugation (2000 rpm, 5 min, 4°C). Digestion was quenched using DPBS containing 2% Penicillin-Streptomycin-Amphotericin B solution. The suspension was filtered through a 100 μm cell strainer, and crypt-containing fractions were collected. Crypts were pelleted, resuspended in DPBS, and quantified microscopically. After centrifugation, crypts were resuspended in organoid growth medium (Hangzhou, China) at 8–10 crypts/μl and embedded in Matrigel^®^ (Corning, USA) droplets (50 μl/well). Polymerization was achieved by incubating plates at 37 °C for 5 min, followed by inverted placement for 25 min. Each well was supplemented with 500 μL prewarmed medium and cultured at 37°C and 5% CO_2_, with medium replacement every 3 days.

### 2.16 Histopathological analysis

The colonic tissues and organoids were fixed in 4% paraformaldehyde at 4°C overnight and subjected to paraffin embedding and slicing. Both paraffin-embedded mouse colon tissues (fixed in 4% paraformaldehyde) and colonic organoids were processed for hematoxylin and eosin (H&E) staining using standardized protocols. Tissue sections (5 μm thickness) and organoid samples underwent parallel processing involving deparaffinization, rehydration, and conventional H&E staining with differentiation/bluing steps, followed by dehydration, xylene clearing, and resin mounting. Histopathological scoring was performed according to established criteria (25).

### 2.17 Immunohistochemistry and immunofluorescence

For immunohistochemistry, sections were deparaffinized in xylene and rehydrated through a graded ethanol series. Antigen retrieval was performed in EDTA buffer (pH 9.0) using microwave heating. After blocking endogenous peroxidase activity with 3% H_2_O_2_ and nonspecific binding with 3% serum, sections were incubated with primary antibody overnight at 4°C. Following PBS washes, HRP-conjugated secondary antibody was applied for 1 h at 37°C. DAB was used for color development, with reaction time monitored microscopically. Counterstaining was performed with hematoxylin, and slides were dehydrated through an ethanol-xylene series before mounting.

For immunofluorescence, sections were deparaffinized in xylene and rehydrated through graded ethanol. After PBS washing, antigen retrieval was performed in EDTA buffer using microwave heating. Sections were permeabilized with 0.3% Triton X-100, blocked with 3% H_2_O_2_ and 3% goat serum, then incubated with primary antibody overnight at 4°C. After washing, sections were incubated with species-matched secondary antibody for 1 h at room temperature. Nuclei were counterstained with DAPI before mounting with antifade medium.

### 2.18 Quantitative real-time PCR analysis

Total RNA was extracted from organoids by a Trizol-bead-based RNA extraction kit (Genstone Biotech, China). cDNA was synthesized using a reverse transcription kit (Thermo Fisher Scientific, USA). Then, qRT-PCR amplification was performed on an iQ5 Real-Time PCR using 2× TB Green^®^ Premix Ex Taq (TakaraBio, China). The relative mRNA expression of genes was calculated using the 2‐ΔΔCt method.

### 2.19 Elisa

The expression of IL-4, IL-13 and IL-25 in the mouse colon organoids was determined using ELISA kits according to the manufacturer’s instructions.

### 2.20 Flow cytometry analysis

Mouse colon organoids were dissociated from Matrigel^®^ (Corning, USA), followed by enzymatic digestion with intestinal tissue digestion solution (Hangzhou, China) at 37 °C for 20 min to generate single-cell suspensions. Cells were filtered through a 70 μm strainer and washed twice with DPBS containing 2% FBS. For marker staining, cells (5×10^5^/ml) were incubated with fluorochrome-conjugated antibodies for 30 min at 4°C. Samples were analyzed on flow cytometer, and data were processed with FlowJo software (v10.7).

### 2.21 Statistical analysis

All data were analyzed using GraphPad Prism 8.0 and are presented as mean ± standard deviation (SD). Statistical comparisons were performed using one-way analysis of variance (ANOVA) or Student’s t-test, as appropriate. A p-value < 0.05 was considered statistically significant.

## 3 Results

### 3.1 Identification prototype blood-absorbed components of BDS

**Figure 1:**
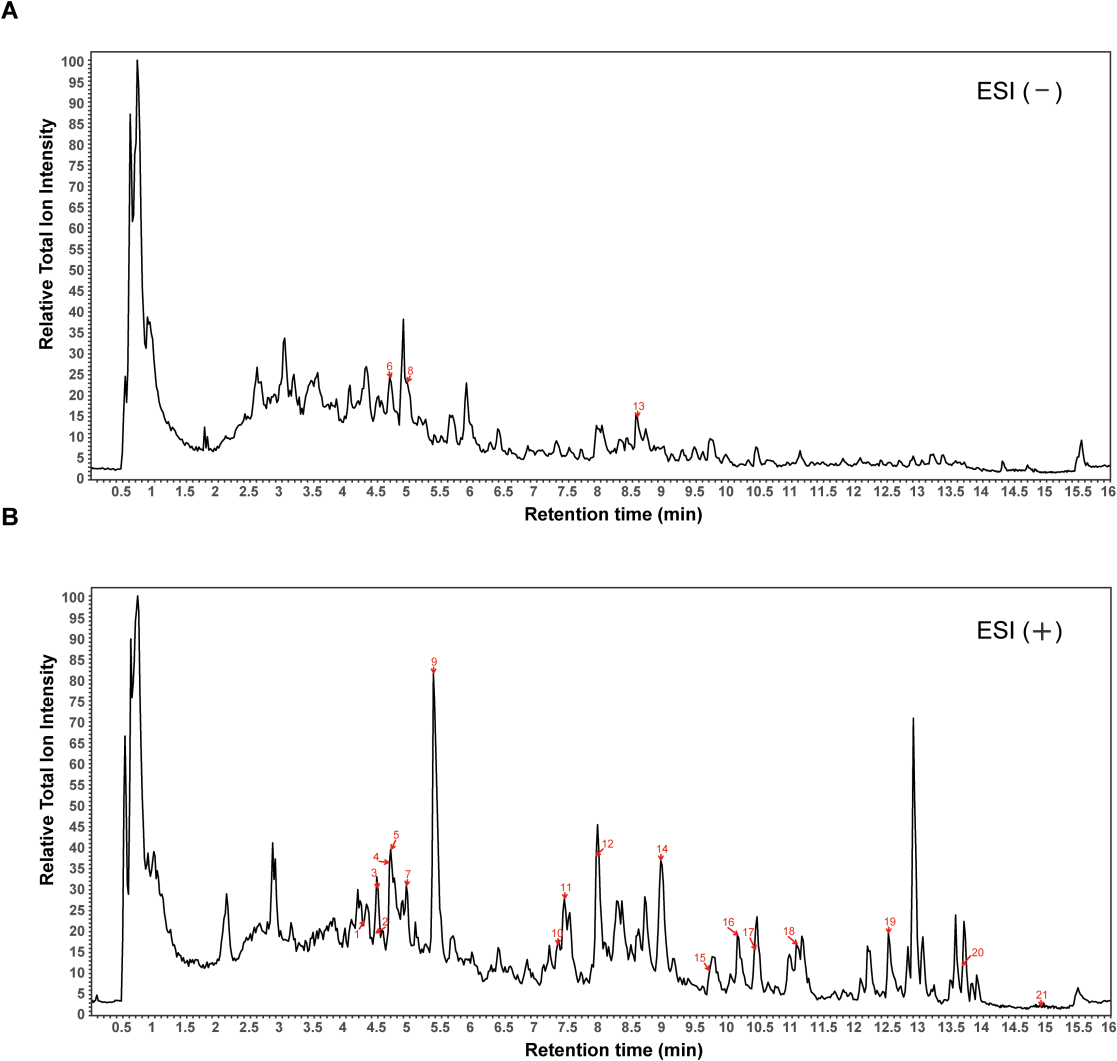
Total ion chromatograms of BDS by UPLC-Q-Exactive MS/MS. **(A)** negative ion mode. **(B)** positive ion mode. The numerical labels in the TIC chromatograms correspond to the compound numbers listed in **Table 1**.

The UPLC-Q-Exactive MS/MS method was established after optimization of chromatographic and mass spectrometric conditions. The sample was analyzed in both positive and negative ion modes to obtain total ion chromatograms (**Figure 1** and **Supplementary Figure 1**). Based on mass-to-charge ratio (*m/z*), retention time, and fragmentation patterns, we identified 21 prototype blood-absorbed components. Their sources were confirmed by comparison with previous studies. Detailed information of these components is listed in **Table 2**.

**Table 2:** Identification of prototype components from BDS in serum.

NO.	RT (min)	Compounds	Formula	m/z	Error/ppm	Ion Mode	Source
1	4.29	Isoliquiritigenin	C_15_H_12_O_4_	257.0809	0.06	M+H	GC
2	4.46	Marmesin	C_14_H_14_O_4_	247.0966	0.40	M+H	QH, DH, HQ
3	4.50	Nodakenin	C_20_H_24_O_9_	409.1494	0.32	M+H	QH, DH, HQ
4	4.69	Naringenin	C_15_H_12_O_5_	273.0757	-0.03	M+H	QH, BH
5	4.71	Lomatin	C_14_H_14_O_4_	247.0966	0.43	M+H	HQ
6	4.71	Naringenin 7-rhamnoglucoside	C_27_H_32_O_14_	579.1729	1.75	M-H	ZK, GC
7	4.95	Hesperetin	C_16_H_14_O_6_	303.0863	0.07	M+H	ZK
8	4.97	Neohesperidin	C_28_H_34_O_15_	609.1835	1.72	M-H	ZK
9	5.37	Auraptenol	C_15_H_16_O_4_	261.1121	-0.06	M+H	CH, QH
10	7.33	Methoxsalen	C_12_H_8_O_4_	217.0496	0.35	M+H	DH, HQ
11	7.43	Beta-lapachone	C_15_H_14_O_3_	243.1016	0.15	M+H	SJ
12	7.94	Angelol b	C_20_H_24_O_7_	377.1595	0.04	M+H	DH, JG
13	8.58	9,12,13-Todea	C_18_H_34_O_5_	329.2337	1.19	M-H	FL
14	8.94	Isomeranzin	C_15_H_16_O_4_	261.1122	0.30	M+H	JG
15	9.72	Glycyrrhizinic acid	C_42_H_62_O_16_	823.4113	0.27	M+H	GC
16	10.16	Nobiletin	C_21_H_22_O_8_	403.1389	0.50	M+H	ZK, JG
17	10.43	Chrysophanic acid	C_15_H_10_O_4_	287.0914	-0.10	M+CH3OH+H	BH, CX
18	11.09	5-Hydroxy-3,6,7,8,3’,4’-hexamethoxyflavone	C_21_H_22_O_9_	419.1337	0.13	M+H	BH, SJ
19	12.51	Osthole	C_15_H_16_O_3_	245.1173	0.37	M+H	DH, HQ
20	13.69	Qianhucoumarin A	C_19_H_20_O_6_	327.1227	0.09	M+H-H2O	HQ
21	14.91	Lecithin	C_42_H_80_NO_8_P	758.5698	0.43	M+H	RS

“RS” represents Panax ginseng; “GC” represents Glycyrrhiza uralensis; “CH” represents Bupleurum chinense; “CX” represents Ligusticum sinense; “HQ” represents Peucedanum praeruptorum; “JG” represents Platycodon grandiflorus; “QH” represents Notopterygium incisum; “DH” represents Heracleum hemsleyanum; “FL” represents Poria cocos; “ZK” represents Citrus aurantium; “BH” represents Mentha canadensis; “SJ” represents Zingiber officinale.

### 3.2 Non-targeted serum metabolomics analysis

**Figure 2:**
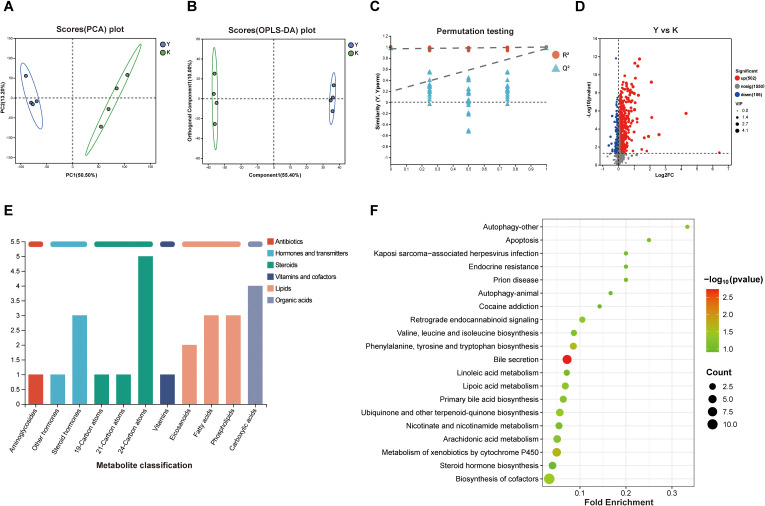
Metabolomic analysis of serum differential metabolites. **(A)** PCA analysis of serum samples (Y: drug-containing serum group; K: blank serum group). **(B)** OPLS-DA analysis of serum samples. **(C)** Permutation test validation of the OPLS-DA model. **(D)** Volcano plot showing differential metabolites between blank serum and drug-containing serum samples. **(E)** Bar plot showing the classification of metabolites. **(F)** KEGG enrichment analysis of differential metabolites.

PCA analysis and OPLS-DA analysis showed that BDS altered serum metabolite profiles in rats (**Figures 2A, B**). The reliability of the OPLS-DA model was validated by 7-cycle interactive validation (**Figure 2C**). Metabolites with VIP>1, P<0.05, and FC>1 in blank serum versus drug-containing serum were selected as differential metabolites. After database matching (PubChem, HMDB) and exclusion of endogenous metabolites, 608 differential metabolites were identified. The volcano plot displays metabolites with significant contributions to intergroup differences (**Figure 2D**). These differential metabolites were primarily enriched in steroid and lipid categories (**Figure 2E**). Metabolic pathway annotation and enrichment analysis revealed that the differential metabolites were primarily associated with the autophagy, apoptosis, bile secretion, linoleic acid metabolism, lipoic acid metabolism, primary bile acid biosynthesis, arachidonic acid metabolism and biosynthesis of cofactors metabolic pathways (**Figure 2F**).

### 3.3 Network pharmacology analysis and molecular docking

**Figure 3:**
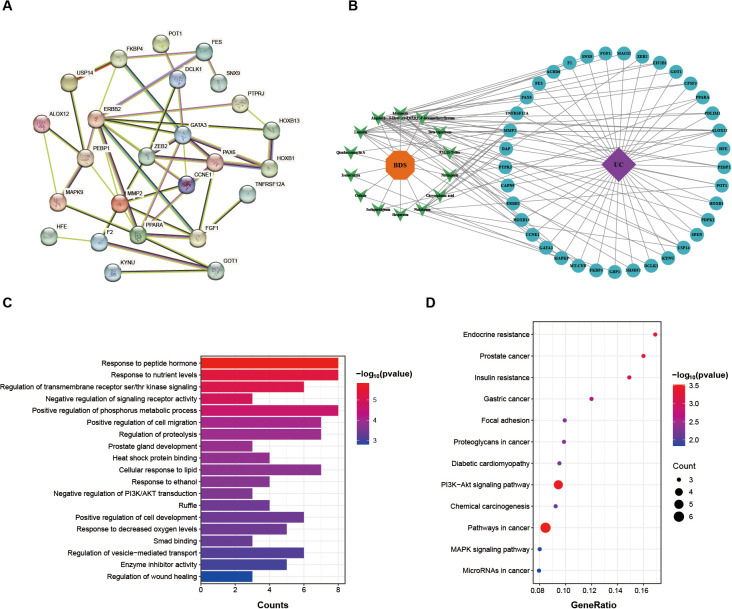
Network pharmacology analysis of prototype blood-absorbed components of BDS. **(A)** Protein-protein interaction (PPI) network of the targets. **(B)** Compound- disease-target network between the interaction of 37 UC-related core genes (blue roundness) and 14 absorbed components (green “V”) of BDS. **(C)** The top 20 enriched GO terms of potential targets of BDS prototype blood-absorbed components. **(D)** KEGG enrichment analysis for the overlapping targets of BDS prototype blood-absorbed components and UC.

A total of 337 DPCs targets were identified from TCMSP and SwissTargetPrediction databases, while 11,723 UC-related targets were retrieved from disease databases (including OMIM, DisGeNET and GeneCards). STRING database was subsequently used to construct a protein-protein interaction (PPI) network of overlapping targets, comprising 37 nodes and 83 edges (**Figure 3A**). The network was further analyzed in Cytoscape 3.10.3 software to visualize the compound-target-disease interactions (**Figure 3B**).

**Figure 4:**
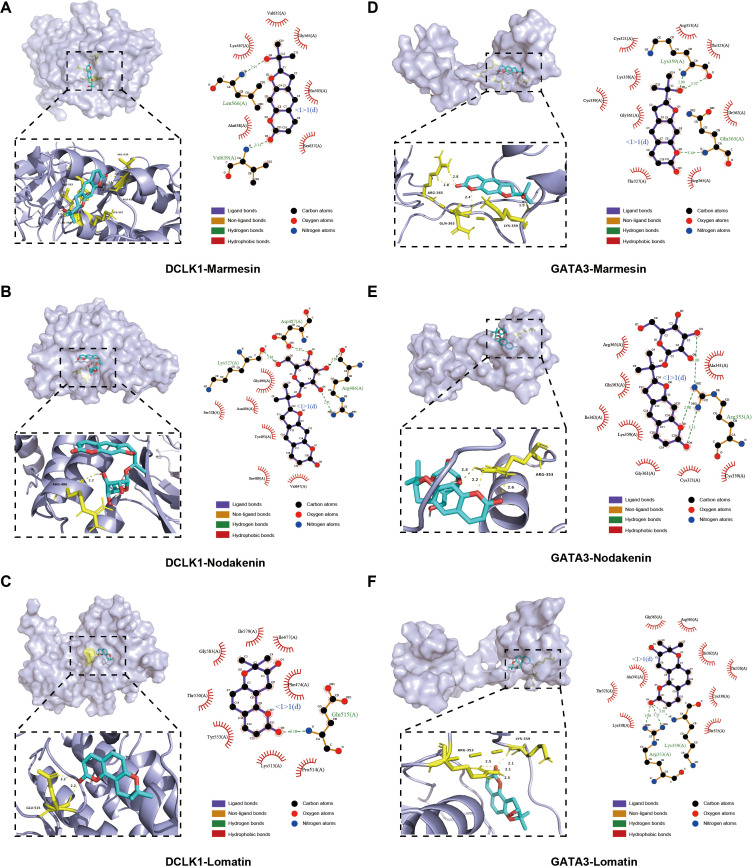
Molecular docking of BDS components to core targets. **(A-C)** Marmesin, Nodakenin, Lomatin with DCLK1. **(D-F)** Marmesin, Nodakenin, Lomatin with GATA3.

GO enrichment analysis showed the TOP 20 significantly enriched items, which mainly involved regulation of wound healing, positive regulation of cell development, negative regulation of PI3K/AKT transduction and cellular response to lipid (**Figure 3C**). KEGG analysis indicated that the overlapping genes were enriched in pathways such as PI3K-AKT and MAPK signaling, as well as several cancer-related pathways. These enriched pathways suggest that BDS-associated targets participate in cellular growth, survival, and stress-response processes, rather than indicating strong specificity toward inflammatory pathways. (**Figure 3D**). Core target screening identified the binding energies of molecular docking between five core targets and the three most promising bioactive compounds (**Supplementary Figure 2**). The six protein-ligand complexes with the lowest binding energies were selected for visualization of the docking results. Schematic diagrams of the most stable binding sites for the six complexes are shown in **Figures 4A–F**.

### 3.4 Single-cell landscape of DSS-induced colon injury and BDS treatment

**Figure 5:**
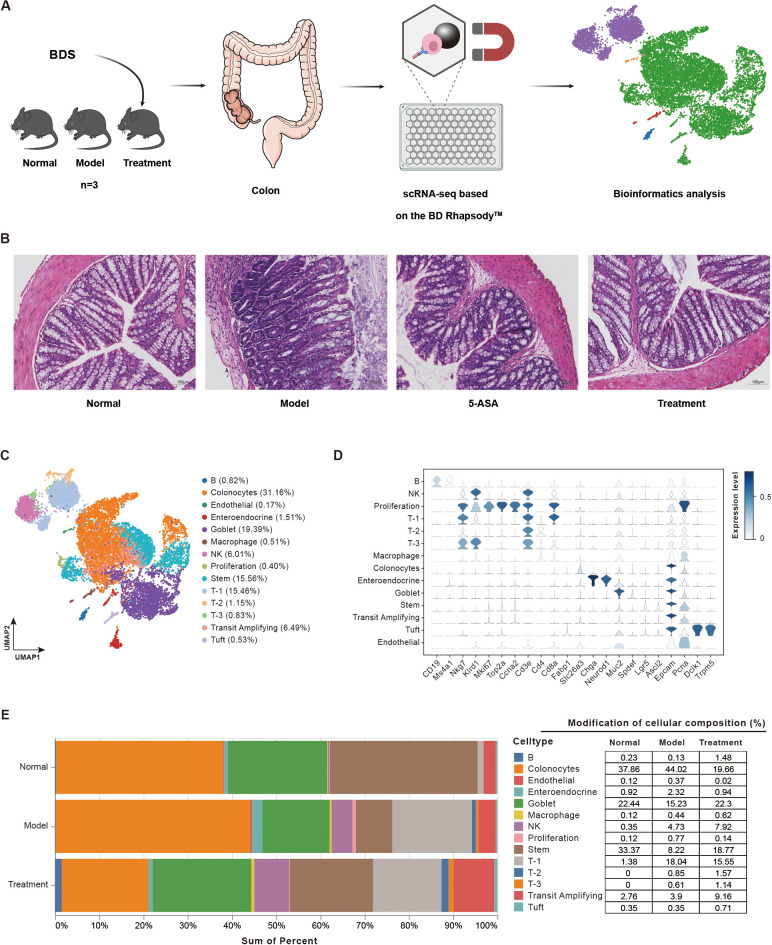
Experimental pipeline and single-cell landscape of BDS treatment in UC mice. **(A)** Flowchart of the Experimental Procedure. **(B)** H&E staining displaying pathological changes in the murine colon under experimental conditions. (Scale bar = 100 µm; 5-ASA, 5−aminosalicylic acid). **(C)** Cell types of sequencing cells projecting on UMAP visualization. Each dot represents a single cell, colored according to its assigned cluster identity, with the respective proportion in parentheses. **(D)** Heatmap of marker genes corresponding to the respective cell types. **(E)** Bar plot showing the cellular composition in three groups (Normal, Model, and Treatment). The adjacent table provides detailed percentages.

To investigate the therapeutic effects of BDS on UC, we performed single-cell RNA sequencing (scRNA-seq) analysis on colon tissues, which were obtained from normal mice (Normal group), DSS-induced UC mice (Model group), and BDS-treated UC mice (Treatment group), each pooled biological sample comprised tissues from 3 mice per group. Due to pooling prior to library construction, mouse-level biological variability could not be assessed, and differential analyses were performed at the cell-population level. **Figure 5A** illustrates the experimental workflow. H&E staining showed significant colonic mucosal damage in the Model group, characterized by disrupted epithelial architecture, crypt loss, and goblet cells depletion. Notably, BDS treatment partially ameliorated these histopathological alterations in UC mice (**Figure 5B**).

Following the methodology described, we processed the integrated single-cell RNA dataset and visualized the results in UMAP (**Figure 5C**). Initial clustering identified 14 distinct cell clusters, 12 of which were robustly annotated based on canonical marker genes, including tuft cells (*Dclk1*, *Trpm5*), transit-amplifying cells (*Epcam*, *Mki67*, *Pcna*), stem cells (*Ascl2*, *Lgr5*), goblet cells (*Muc2*, *Spdef*, *Tff3*), enteroendocrine cells (*Chga*, *Chgb*, *Neurod1*, *Pcsk1*), colonocytes cells (*Fabp1*, *Slc26a3*), B cells (*Cd19*, *Ms4a1*), NK cells (*Il2rb*, *Klrd1*, *Nkg7*), proliferation cells (*Mki67*, *Top2a*, *Ube2c*), and T-1~T-3 cells (*Cd8b1*, *Gzmb*, *Cd4*, *Foxp3*, *Ptprc*) (**Figure 5D**). The remaining two populations, endothelial cells and macrophages, were excluded from downstream analysis due to their indistinct transcriptional profiles with substantial marker gene overlap with T-cell clusters, and failure to meet our stringent quality threshold requiring at least three unique signature genes (logFC>1.5 detected in >30% of cluster cells). Compared to the Normal group, the Model group exhibited a marked reduction in the proportions of goblet cells and stem cells, consistent with the crypt loss and goblet cell depletion observed in H&E staining of colon tissues (**Figure 5E**). In contrast, the proportions of colonocytes, transient amplifying cells, and T cell clusters 1–3 were increased in the Model group. Notably, BDS treatment partially restored these alterations, increasing the proportions of goblet cells, stem cells, and tuft cells.

### 3.5 BDS treatment rebalances colonic epithelial cell subclusters in UC mice

**Figure 6:**
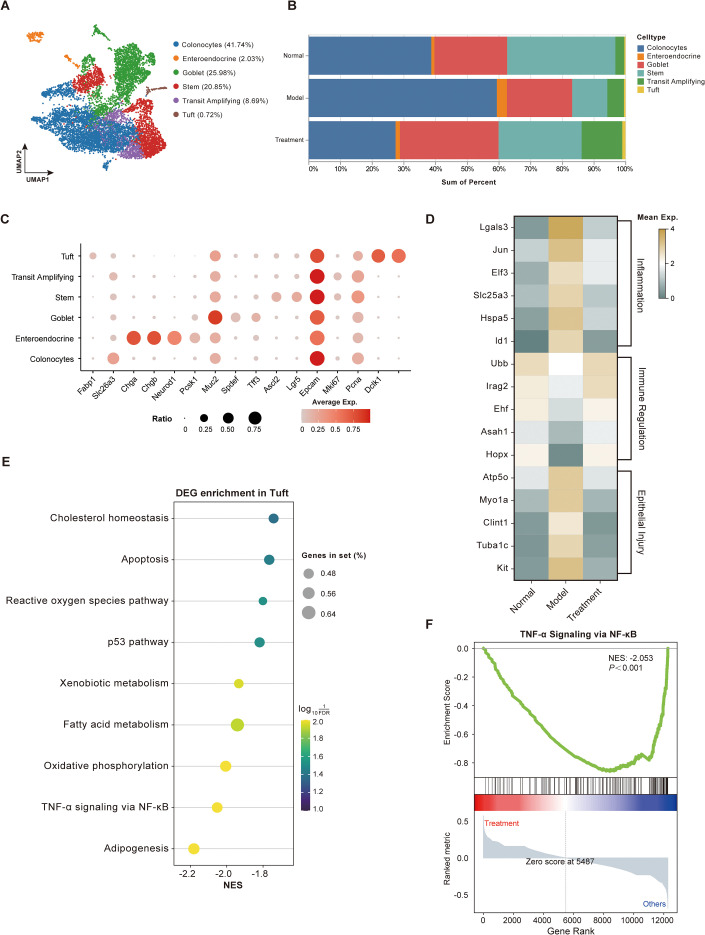
BDS treatment rebalances colonic epithelial cell subclusters in UC mice. **(A)** UMAP visualization of re-clustered epithelial cell subclusters, dividing into 6 subclusters, namely Colonocytes, Enteroendocrine, Goblet, Stem, Transit Amplifying and Tuft. **(B)** Bar plot showing the cellular composition of re-clustered epithelial cell subclusters in three groups (Normal, Model, and Treatment). **(C)** Bubble plot representing the expression levels of manually annotated classical marker genes. **(D)** Heatmap showing the expression levels of genes associated with inflammation, immune responses and injury genes in Tuft cells. **(E)** Bubble plot depicting the enrichment pathways of differentially expressed genes (DEGs) in BDS-treated Tuft cells. **(F)** GSEA analysis of differentially expressed genes in TNF-α Signaling via NF-κB pathway in BDS-treated tuft cells. Single-cell data were generated from pooled samples (n = 3). DEG identification used Seurat’s Wilcoxon test with adjusted *p* < 0.05.

Colonic epithelial populations play pivotal roles in maintaining intestinal barrier integrity and mucosal homeostasis, with their dysregulation constituting a basic pathological hallmark of UC (26). To systematically characterize BDS-mediated effects, we performed subset-specific re-clustering of epithelial cells using well-established marker genes, identifying six transcriptionally distinct subclusters (**Figures 6A, B**). All subclusters uniformly expressed the canonical pan-epithelial marker Epcam while maintaining subcluster-specific gene expression profiles, validating the robustness of our clustering approach (**Figure 6C**).

Within the tuft cell subcluster, BDS treatment was associated with coordinated transcriptional changes. Specifically, genes related to inflammatory responses and epithelial injury exhibited reduced expression, whereas genes associated with immune-related processes showed increased expression in the BDS-treated group (**Figure 6D**). Pathway enrichment analysis of tuft cell–associated differentially expressed genes revealed predominant involvement of cholesterol homeostasis, fatty acid metabolism, and stress-response pathways, including apoptosis, reactive oxygen species, and p53 signaling (**Figure 6E**). Gene set enrichment analysis further indicated altered activity of the TNF-α signaling pathway via NF-κB in tuft cells following BDS treatment (**Figure 6F**). Notably, several of these metabolic- and immune-related pathways are consistent with those highlighted by the metabolomics and network pharmacology analyses, suggesting concordant pathway-level changes across multiple analytical platforms.

### 3.6 BDS treatment ameliorates colitis-associated immune dysregulation

**Figure 7:**
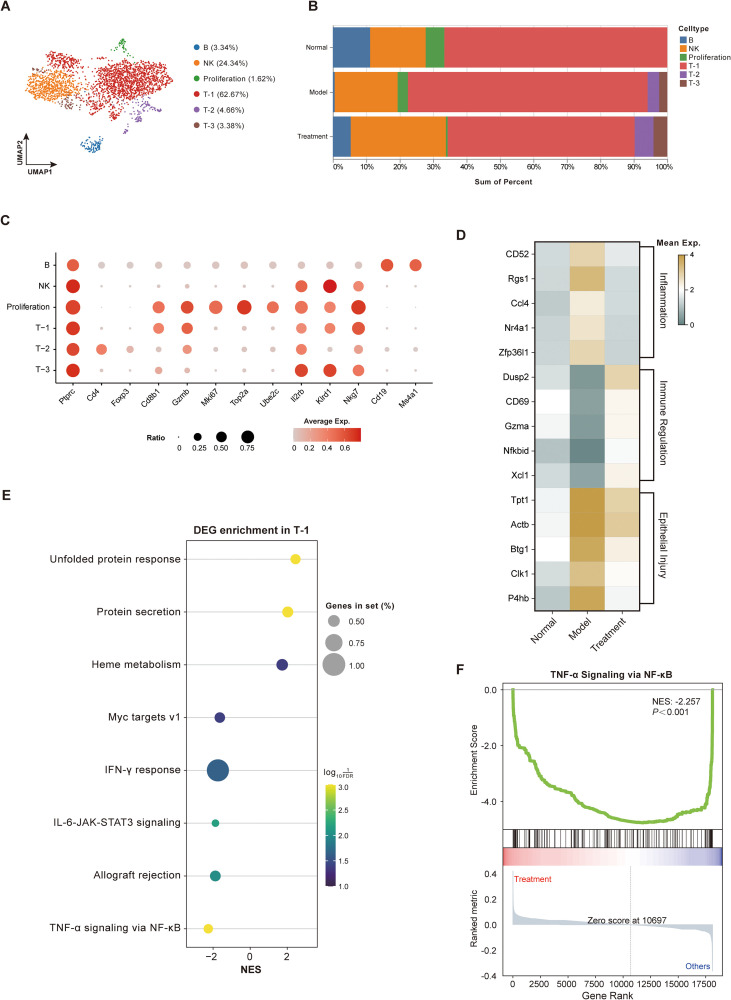
BDS treatment ameliorates immune dysregulation in UC mice. **(A)** UMAP visualization displaying re-clustering of T lymphocyte, B lymphocyte, and NK cells, revealing 6 subclusters namely B, NK, Proliferation, T-1, T-2 and T-3. **(B)** Bar plot showing the cellular composition of re-clustered immune cell subclusters in three groups (Normal, Model, and Treatment). **(C)** Bubble plot representing the expression levels of manually annotated classical marker genes. **(D)** Heatmap showing the expression levels of genes associated with inflammation, immune responses and injury genes in T-1 cells. **(E)** Bubble plot depicting the enrichment pathways of differentially expressed genes (DEGs) in BDS-treated T-1 cells. **(F)** GSEA analysis of differentially expressed genes in TNF-α Signaling via NF-κB pathway in BDS-treated T-1 cells.

Lymphocyte T, В cells and natural killer (NK) cells play critical roles in innate and adaptive immunity. We re-clustered the immune cells from our dataset into six subclusters—B, NK, Proliferation, T-1, T-2, and T-3—based on canonical markers and cluster-specific genes (**Figure 7A**). Interestingly, T-2 and T-3 were detected only in the Model and Treatment groups, and BDS treatment significantly increased their proportions while concomitantly decreasing the T-1 (**Figure 7B**). We also observed distinct changes in the proportions of B cells of Model mice that were subsequently reversed by BDS treatment, while remaining subtypes exhibited minor changes. Notably, T-1 cells displayed characteristic CD8^+^ features, while T-2 cells exhibited a distinct CD4^+^ phenotype (**Figure 7C**). Further, BDS treatment downregulated genes associated with inflammation and epithelial injury in T-1 cells and, conversely, upregulated genes involved in immune responses (**Figure 7D**). Pathway enrichment analysis of differentially expressed genes in T-1 cells revealed involvement of the unfolded protein response, heme metabolism, IFN-γ response, and IL-6–JAK–STAT3 signaling pathways (**Figure 7E**). Gene set enrichment analysis further demonstrated that BDS treatment markedly suppressed NF-κB-mediated TNF-α signaling axis in T-1 cells (**Figure 7F**). Notably, several of these immune-related pathways are consistent with the immune regulatory processes predicted by the network pharmacology analysis, suggesting a convergence between system-level pathway predictions and immune cell–specific transcriptional responses.

### 3.7 BDS modulates intercellular communication in mouse colon with UC

**Figure 8:**
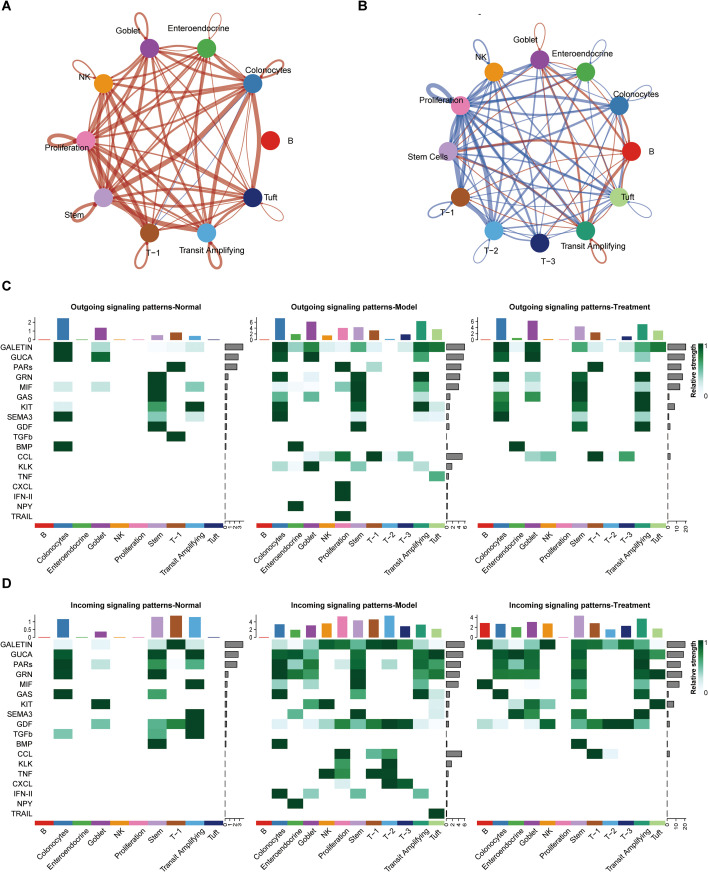
BDS-mediated cell communication network analysis. **(A)** Overall networks of intercellular crosstalk among cell types in Normal *vs.* Model groups. **(B)** Overall networks of intercellular crosstalk among cell types in Model *vs.* Treatment groups. Each node represents a distinct cell type, and the thickness of connecting lines is proportional to the number of interactions between two cell types. Red lines denote increased communication intensity, while blue lines indicate decreased communication intensity. **(C)** Heatmap showing the outgoing communication patterns across different cell types in Normal, Model, and Treatment groups. **(D)** Heatmap showing the incoming communication patterns across different cell types in Normal, Model, and Treatment groups. The relative strength of each signal pathway is color coded form gray to green.

To elucidate how BDS treatment modulates intercellular communication in UC mice, we performed systematic comparative analyses of cell-cell interaction networks across Normal, Model, and Treatment groups. CellChat analysis revealed a pronounced enhancement of global communication networks in Model versus Normal groups, particularly manifested through intensified immune cell interactions (**Figures 8A, B** and **Supplementary Figures 3A, B**). After BDS treatment, we observed a relative attenuation of immune cell–dominated communication patterns, accompanied by partial restoration of epithelial cell signaling networks. These findings demonstrate that BDS treatment effectively rebalances the pathological hypercommunication state characteristic of UC, preferentially normalizing epithelial interactions while suppressing excessive immune cell crosstalk. To further delineate key intercellular communication events, we specifically interrogated signaling from tuft and T-1 subclusters. Notably, compared with Normal controls, the Model group exhibited enhanced outgoing signals from tuft cells to multiple recipient clusters, including T-1 cells, NK cells, and other immune subsets (**Supplementary Figures 3C, D**). BDS treatment significantly attenuated these tuft cell-mediated interactions. Conversely, T-1 demonstrated opposing communication patterns, showing increased connectivity with neighboring cell types post-treatment. **Supplementary Figure 4** displays the overall ligand-receptor interaction probability heatmap.

Analysis of outgoing and incoming signaling patterns revealed broadly altered intercellular communication across the Normal, Model, and Treatment groups. In model group, both epithelial and immune cell populations exhibited increased communication activity, indicating a dysregulated signaling environment. Among these changes, tuft cells showed relatively enhanced signaling involving pathways such as TGF-β, Kit, and SEMA3, together with increased reception of inflammation-associated inputs, while T-1 cells displayed elevated chemokine-related signaling. Following BDS treatment, the overall communication landscape shifted toward normalization, characterized by reduced inflammatory-associated inputs and partial restoration of homeostatic signaling patterns. These observations suggest that BDS modulates colonic intercellular communication, with tuft cells and T-1 cells serving as representative examples of broader communication changes rather than exclusive regulatory centers (**Figures 8C, D**).

### 3.8 BDS promotes colon organoid regeneration through tuft cell expansion and type 2 immune activation

**Figure 9:**
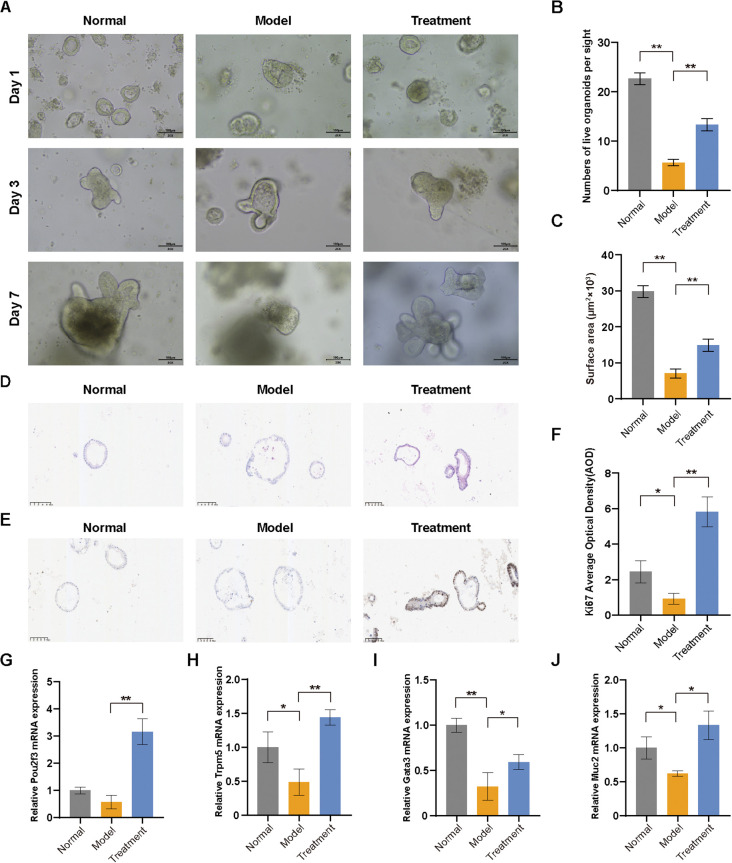
BDS stimulates colon organoid expansion and alleviates UC-mediated tissue damage. **(A)** The growth status of the colon organoids was observed under a microscope (scale bar = 100 μm). **(B)** Number of viable organoids per field of view. **(C)** The size of organoid surface area. **(D)** H&E staining of colon organoids. **(E)** Immunohistochemical staining for Ki67 in colon organoids. **(F)** Quantitative analysis of Ki67 expression by AOD measurement. Relative mRNA level of **(G)** Pou2f3, **(H)** Trpm5, **(I)** Gata3, **(J)** Muc2. Data are presented as the mean ± SD, *n* = 3. Significance levels are indicated as ^*^p < 0.05, ^**^p < 0.01.

We established colon organoids to investigate BDS therapeutic effects. On day 1, all groups exhibited characteristic spherical or cyst-like structures. On day 3, UC microenvironment-induced structural damage became evident in model group, manifesting as disorganized architecture with inflammatory features. On day 7, UC organoids showed significantly decreased the number of live organoids and the surface area per organoid, while BDS treatment substantially attenuated these degenerative changes and promoted organoid regeneration (**Figures 9A–C**). H&E staining showed that normal organoids were uniformly spherical with a single intact lumen and continuous epithelium, UC organoids displayed irregular shapes, lumen collapse, epithelial detachment and debris (**Figure 9D**); BDS treatment largely restored epithelial integrity and lumen uniformity; Ki67 immunohistochemistry further confirmed that BDS-treated organoids exhibited active proliferative activity (**Figures 9E, F**). In addition, UC organoids showed reduced *pou2f3*, *trpm5*, *gata3*, and *muc2* expression, which was significantly reversed by BDS treatment (**Figures 9G–J**).

**Figure 10:**
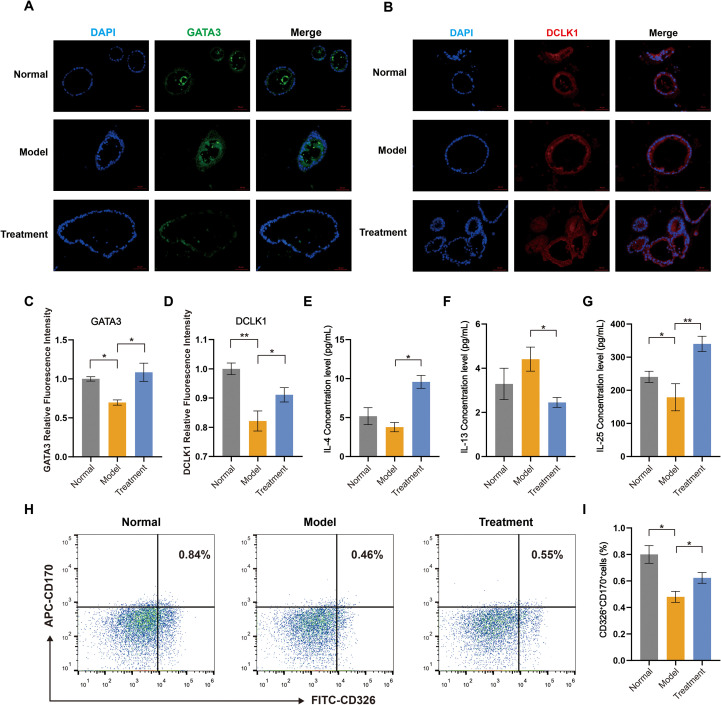
BDS treatment increases tuft cell numbers and promotes type 2 immune cytokine release. **(A)** Representative images of immunofluorescence for GATA3 (scale bar = 50 μm). **(B)** Representative images of immunofluorescence for DCLK1 (scale bar = 50 μm). **(C)** Relative fluorescence intensity of GATA3. **(D)** Relative fluorescence intensity of DCLK1. **(E)** ELISA measurement of IL-4 concentration in colon organoids. **(F)** ELISA measurement of IL-13 concentration in colon organoids. **(G)** ELISA measurement of IL-25 concentration in colon organoids. **(H)** Flow cytometry analysis of tuft cell proportions in colon organoids. **(I)** The percentage of CD170^+^CD326^+^ Tuft cells in colon organoids. Data are presented as the mean ± SD, *n* = 3. Significance levels are indicated as ^*^p < 0.05, ^**^p < 0.01.

The distribution patterns of DCLK1 and GATA3 in colon organoids were further visualized by immunofluorescence staining (**Figures 10A, B**). Quantitative analysis demonstrated that BDS treatment significantly rescued the UC microenvironment-induced reduction in both DCLK1 and GATA3 expression, as confirmed by the increased relative fluorescence intensity (**Figures 10C, D**). BDS treatment increased IL-4 and IL-25 but decreased IL-13 in colon organoids (**Figures 10E–G**). Flow cytometry demonstrated a marked decrease in tuft cell frequency in Model group, which was reversed by BDS treatment, with CD326^+^CD170^+^ tuft cells increasing (**Figures 10H, I** and **Supplementary Figure 5**).

## 4 Discussion

In this study, we systematically explored the multidimensional mechanisms by which BDS treats UC by integrating metabolomics, network pharmacology, molecular docking and single-cell transcriptomic analyses. BDS modulated serum metabolite profiles dominated by steroids and lipids, thereby influencing steroid hormone biosynthesis, bile acid metabolism, and lipid pathways such as arachidonic acid and linoleic acid metabolism. Network pharmacology further confirmed BDS’s critical roles in regulating inflammatory responses and cellular proliferation. Molecular docking revealed the most promising target protein-ligand complexes and visualized their favorable binding poses. Single‐cell RNA‐seq analysis revealed that UC microenvironment significantly alters the proportions of colonic epithelial and immune cells, changes that were substantially reversed by BDS treatment. Notably, tuft cells and T-1 cell subclusters emerged as key effector populations mediating BDS’s therapeutic effects, synergistically restoring local immune homeostasis. Complementary *in vitro* organoid experiments demonstrated BDS’s capacity to promote crypt structure regeneration and expand CD326^+^CD170^+^ Tuft cells, while reshaping type 2 immunity through upregulation of IL-4/IL-25 and downregulation of IL-13.

Non-targeted metabolomics identified that BDS predominantly affects lipid-related pathways, especially those involving arachidonic acid and linoleic acid. Metabolites within these pathways—such as prostaglandins, leukotrienes, and lipoxins—serve as key mediators of inflammation. For example, PGE_2_ modulates inflammatory processes by regulating T-cell and macrophage functions (27); leukotrienes exacerbate tissue injury by recruiting neutrophils and amplifying inflammatory responses (28); and lipoxins help terminate inflammation and promote tissue repair by inhibiting neutrophil migration, thereby maintaining dynamic inflammatory balance (29). Moreover, our previous work demonstrated that BDS exerts anti-inflammatory effects by inhibiting the PI3K/AKT/NF-κB signaling pathway and downregulating the expression of inflammatory cytokines such as TNF-α, IL-1β, and IL-6 (19). The consistency between these multi-omics results demonstrated the reliability of this integrated analytical approach in elucidating the pharmacological mechanisms of herbal medicines.

scRNA-seq analysis of colonic cell-type heterogeneity provided new mechanistic insights by confirming that UC induces a decrease in epithelial-cell proportions and an increase in immune–cell proportions—changes that were restored upon BDS treatment. Building on growing evidence that tuft cells represent an epithelial subclusters endowed with both immune–sensing and tissue–repair functions, T and that T-1 cells represent a CD8^+^-biased subcluster derived from the T/NK compartment characterized by high *Cd8b1* and *Gzmb* expression, we reasoned that these two populations play pivotal roles in UC pathogenesis. Given their pronounced shifts in relative abundance and documented functions in mucosal defense and cytotoxic immunity (30, 31), we selected the tuft and T-1 subclusters for deeper interrogation. Accordingly, we profiled their DEGs and pathway alterations to elucidate how BDS modulates their inflammatory and homeostatic signaling networks. We found that during UC pathogenesis, tuft cells exhibited upregulation of genes associated with inflammation and epithelial injury, a pattern that was suppressed by BDS treatment. Notably, after BDS administration, both tuft cells and T-1 cells showed coordinated inhibition of pro-inflammatory pathways—specifically NF-κB–mediated TNF-α signaling—indicating a synergistic role for these subclusters in maintaining mucosal immune homeostasis. CellChat analysis revealed that UC progression enhanced pan-cellular communication, especially among immune cells (Normal vs Model). BDS treatment reversed this trend by globally reducing interaction intensities while selectively restoring epithelial crosstalk (Model vs Treatment). Notably, tuft cells play a central role in the dysregulated crosstalk between epithelial and immune cells, with T-1 cells as secondary contributors. BDS treatment may contribute to this effect by suppressing aberrant inflammatory signaling within these two subsets while promoting the transmission of homeostatic signals such as Kit and GDF.

In the present study, network pharmacology and single-cell transcriptomic analyses were applied as complementary approaches to explore the potential mechanisms of Renshen Baidu San (BDS) in DSS-induced colitis. Network pharmacology provided a systems-level perspective by predicting putative targets and signaling pathways associated with the absorbed components of BDS, highlighting pathways related to cellular metabolism, proliferation, and immune regulation. In contrast, single-cell RNA sequencing enabled cell-type–resolved characterization of transcriptional changes within the colonic microenvironment following BDS treatment. Notably, several pathways and biological processes suggested by network pharmacology exhibited concordant transcriptional alterations at the single-cell level, particularly within epithelial subpopulations such as tuft cells and within specific immune cell subsets, including T-1 cells. This convergence suggests that the predicted molecular networks may be preferentially engaged in distinct cellular contexts rather than uniformly across the tissue. Although these approaches do not establish direct causal relationships, their integration provides mutually supportive evidence linking system-level pathway predictions with cell-specific transcriptional responses. Together, this multi-layered analysis supports an integrative, hypothesis-generating framework in which BDS-associated molecular targets and pathways are reflected in coordinated cellular responses, thereby offering mechanistic insights that warrant further functional validation.

Our *in vitro* organoid studies further demonstrate that BDS treatment effectively repairs UC-induced structural damage, restoring epithelial integrity and luminal uniformity—consistent with its known capacity to enhance tight junction proteins such as Occludin and inhibit apoptosis (17). Notably, BDS promoted the expansion of CD326^+^CD170^+^ Tuft cells, further evidenced by restored expression of *Pou2f3*, *Trpm5*, and *Gata3*—key transcriptional regulators governing tuft cell identity and functionality (6, 32). This tuft cells expansion activated downstream immune networks, characterized by upregulated IL-4/IL-25 and downregulated IL-13, indicating a transition toward a tissue-reparative type 2 immune microenvironment (33, 34). This mechanism parallels β-glucan-induced AhR/IL-22 signaling reported in colitis models (35), yet diverges by emphasizing Tuft cell-derived IL-25-mediated immune cell crosstalk rather than direct microbial modulation. Additionally, our work uniquely identifies BDS as a pharmacological inducer of tuft cell proliferation, contrasting with prior reports focusing on microbial-driven tuft cell activation (5, 36). It is important to note that organoid systems primarily recapitulate epithelial compartments; thus, our validation focused on epithelial differentiation markers and IL-25 secretion, which are the most functionally relevant readouts of tuft-cell involvement in epithelial–immune crosstalk (5, 37). Together, these results—while not exhaustive—provide convergent evidence supporting the contribution of tuft cells to BDS-mediated mucosal repair, and highlight the need for future tuft-cell–specific functional studies.

While this study establishes a comprehensive framework for BDS’s therapeutic mechanisms in UC, several limitations should be noted. First, although our colonic organoid model replicates key UC features—such as lumen collapse and epithelial detachment—it cannot fully capture the complexity of human UC pathophysiology, particularly the dynamic interactions between gut microbiota and immune cells (38). Second, although we identified serum metabolites and signaling pathways modulated by BDS, the precise bioactive constituents driving Tuft cell expansion remain undefined. Future work should employ activity‐guided fractionation or CRISPR‐based functional screens to isolate and validate the critical components within the BDS formula (39). Third, while our focus on Tuft cells and T-1 subsets revealed coordinated anti-inflammatory effects, other immune populations—such as regulatory T cells and ILC2s—may also contribute to BDS’s efficacy (37, 40, 41). We also acknowledge that our current validation does not fully resolve the functional heterogeneity of Tuft cells. Future studies incorporating tuft-cell depletion models, IL-25 pathway blockade, or lineage-tracing approaches will be essential to more comprehensively define their causal contribution to BDS-mediated mucosal healing.

## 5 Conclusion

In summary, our integrated multi-omics and functional analyses suggest that BDS ameliorates UC by reshaping serum lipid and steroid metabolomes, modulating key inflammatory and proliferative pathways, and restoring colonic epithelial–immune balance accompanied by expansion and transcriptional changes in tuft and T-1 cell subclusters. Colon organoid validation provides evidence for BDS’s ability to repair epithelial architecture and promote a tissue-reparative type-2 immune milieu through tuft cell–mediated IL-25 secretion. These findings not only elucidate the mechanistic underpinnings of BDS—a classical TCM formula—in UC therapy but also identify tuft cells as candidate whose dysregulation contributes to disease and whose pharmacological targeting may offer novel therapeutic avenues. Future work aimed at isolating the specific bioactive constituents responsible for these effects will further advance the development of targeted interventions for UC.

## Data Availability

The datasets presented in this study can be found in online repositories. The names of the repository/repositories and accession number(s) can be found below: https://www.ncbi.nlm.nih.gov/, PRJNA1390391.

## Supplementary material

Supplementary Figure 1 Total ion chromatograms of drug-containing serum by UPLC-Q-Exactive MS/MS. **(A)** negative ion mode. **(B)** positive ion mode.

Supplementary Figure 2 Heat map of molecular docking binding energy.

Supplementary Figure 3 BDS-mediated intercellular crosstalk in the mouse colon with UC. **(A)** Heatmap showing differential number of interactions in Normal vs. Model groups. **(B)** Heatmap showing differential number of interactions in Model *vs.* Treatment groups. **(C)** Chord diagram displaying the differential interactions between Tuft and other cell types in Normal, Model, and Treatment groups. **(D)** Chord diagram displaying the differential interactions between T-1 and other cell types in Normal, Model, and Treatment groups.

Supplementary Figure 4 BDS-mediated ligand-receptor interaction probability heatmap.

Supplementary Figure 5 Gating strategy for flow cytometry analysis. Gating was applied consistently to all groups: **(A)** cells were first gated on FSC-A vs SSC-A to exclude debris, **(B)** followed by singlet gating using FSC-H vs FSC-A, and **(C)** target populations (CD326^+^CD170^+^ tuft cells) were identified by subsequent fluorescence staining.
