# Supplementary material for: Renshen-Baidu-San restores epithelial–immune crosstalk and drives type 2 immune repair in ulcerative colitis: an integrated multi-omics study
Source: Front Immunol. 2026 Mar 11;17:1777808. doi: 10.3389/fimmu.2026.1777808 (PMC13012912; doi:10.3389/fimmu.2026.1777808)
Supplement: Supplementary file 2 [file DataSheet2.pdf]

Supplementary material

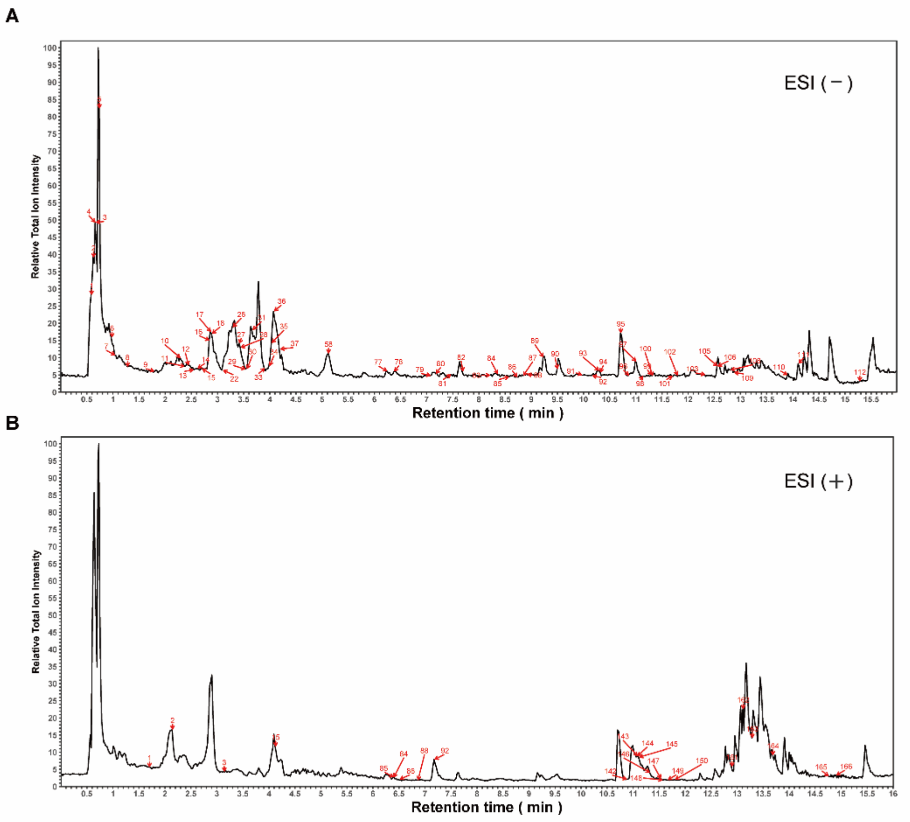

Figure S1. Total ion chromatograms of drug-containing serum by UPLC-Q-Exactive MS/MS. (A) negative ion mode. (B) positive ion mode.

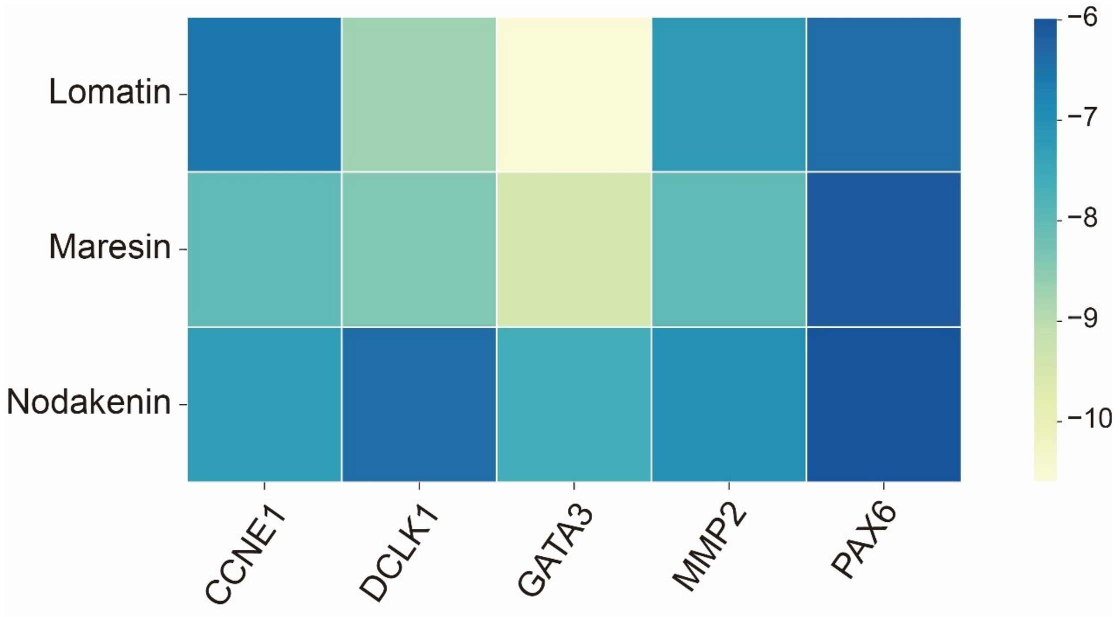

Figure S2. Heat map of molecular docking binding energy.

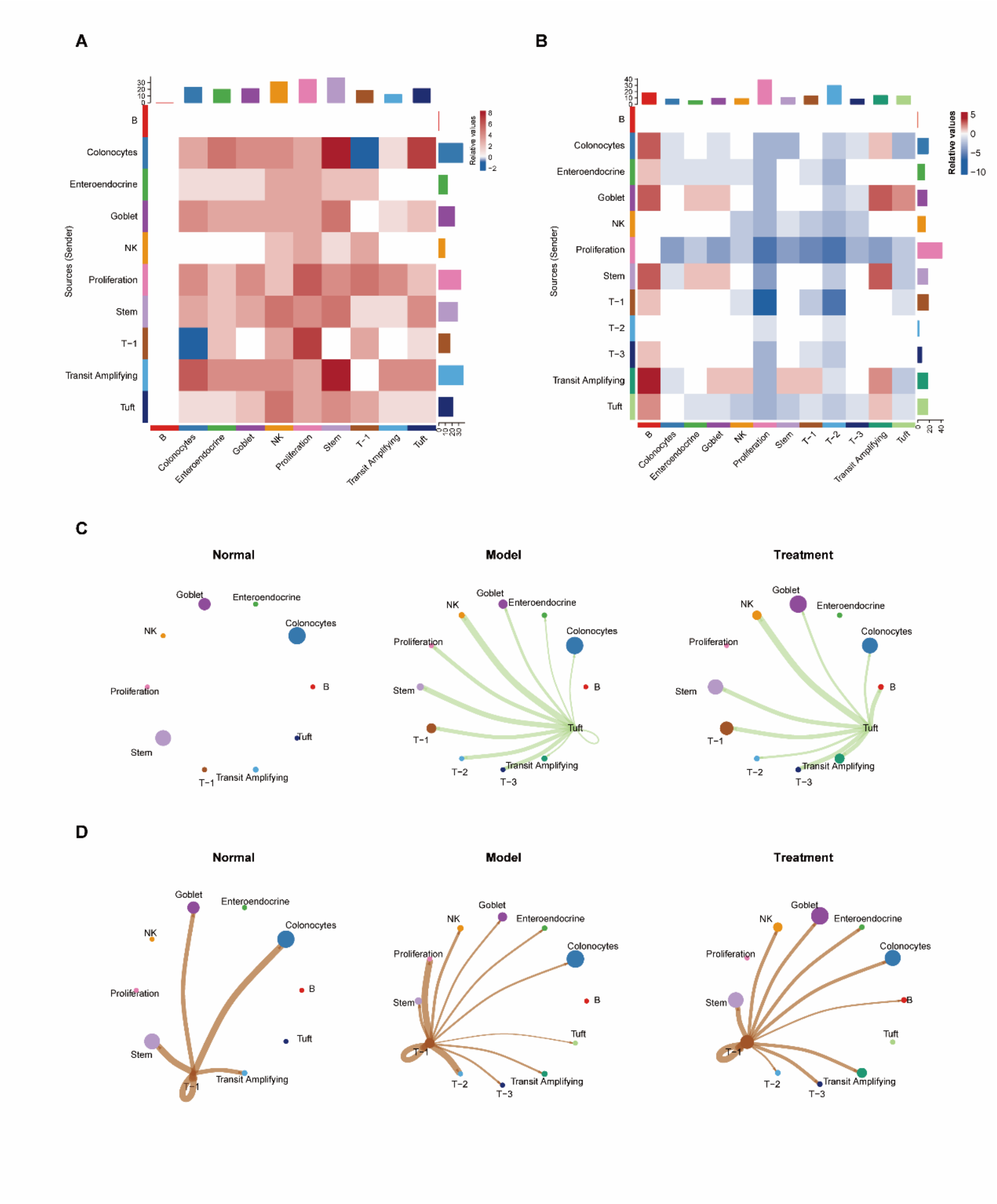

Figure S3. BDS-mediated intercellular crosstalk in the mouse colon with UC. (A) Heatmap showing differential number of interactions in Normal vs. Model groups. (B) Heatmap showing differential number of interactions in Model *vs.* Treatment groups. (C) Chord diagram displaying the differential interactions between Tuft and other cell types in Normal, Model, and Treatment groups. (D) Chord diagram displaying the differential interactions between T-1 and other cell types in Normal, Model, and

Treatment groups

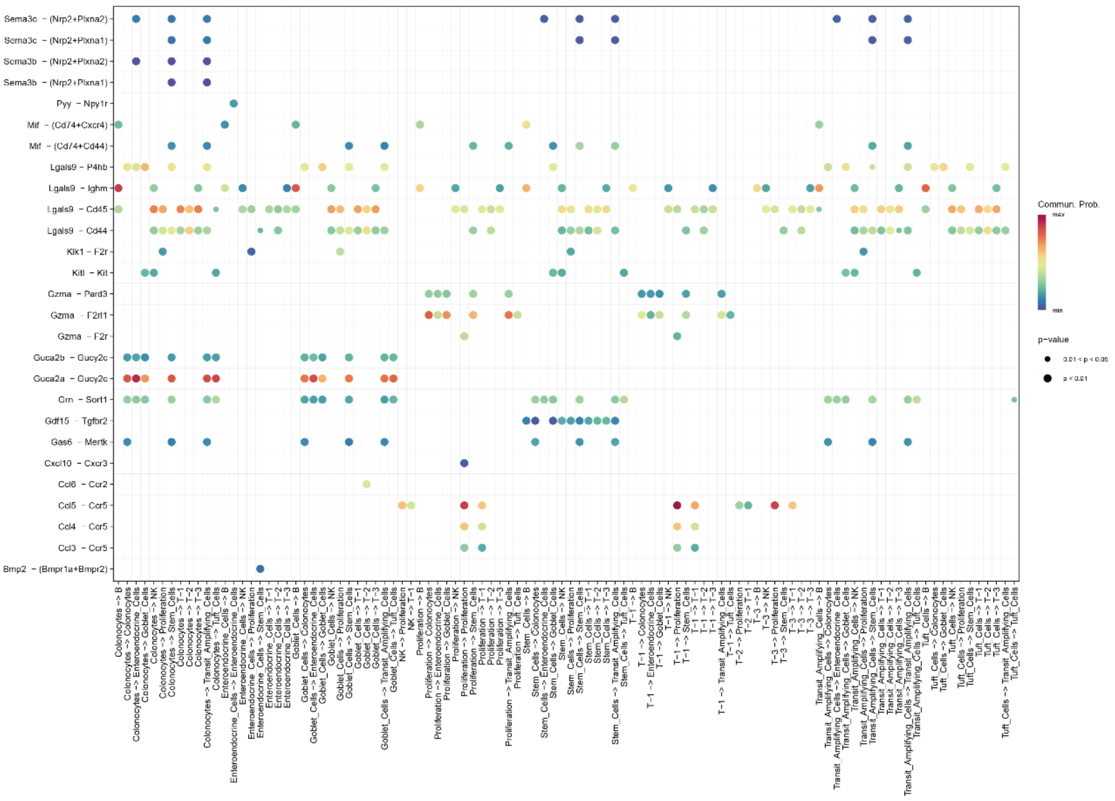

Figure S4. BDS-mediated ligand-receptor interaction probability heatmap.

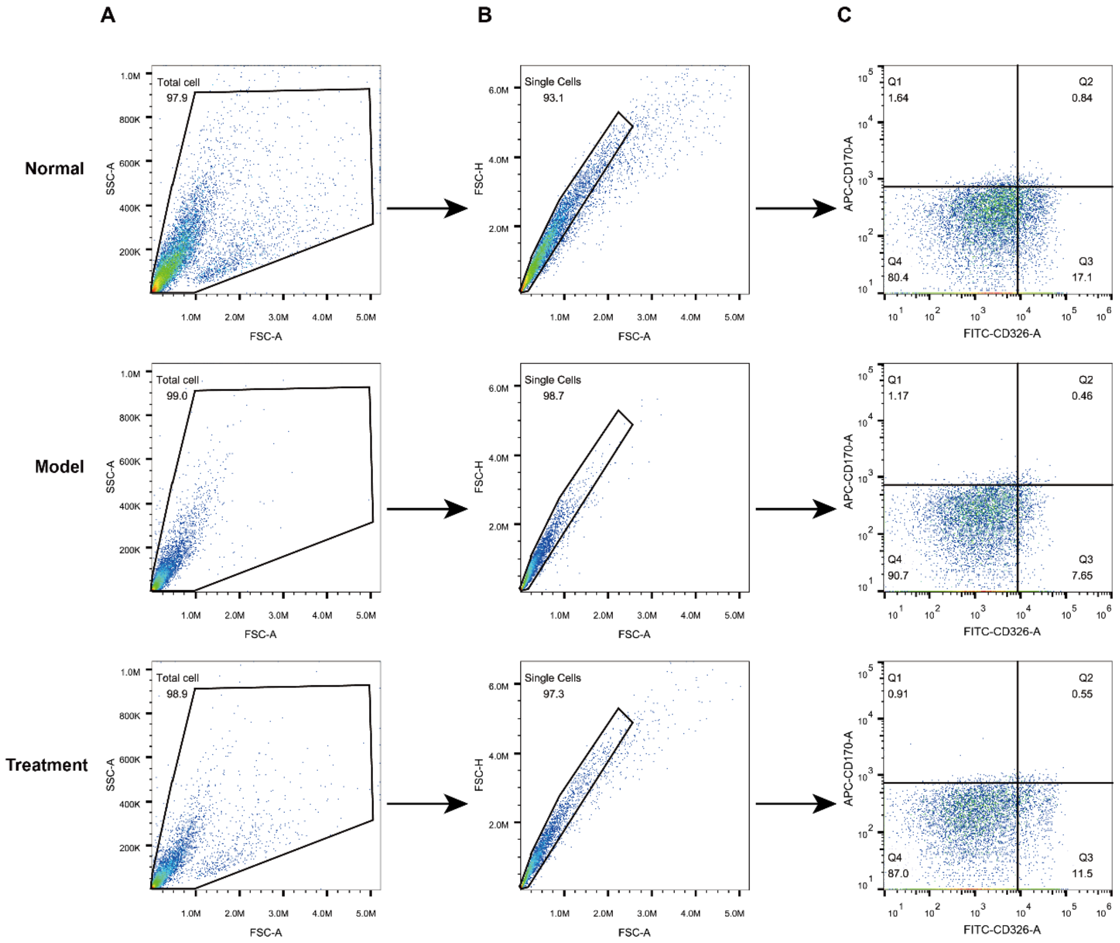

Figure S5. Gating strategy for flow cytometry analysis. Gating was applied consistently to all groups: (A) cells were first gated on FSC-A vs SSC-A to exclude debris, (B) followed by singlet gating using FSC-A vs FSC-H, and (C) target populations ($CD326^{+}CD170^{+}$ tuft cells) were identified by subsequent fluorescence staining
